# Outcomes for Pressure Ulcer Trials (OUTPUTs): protocol for the development of a core domain set for trials evaluating the clinical efficacy or effectiveness of pressure ulcer prevention interventions

**DOI:** 10.1186/s13063-019-3543-9

**Published:** 2019-07-22

**Authors:** Anna Lechner, Jan Kottner, Susanne Coleman, Delia Muir, Heather Bagley, Dimitri Beeckman, Wendy Chaboyer, Janet Cuddigan, Zena Moore, Claudia Rutherford, Jochen Schmitt, Jane Nixon, Katrin Balzer

**Affiliations:** 1 Department of Dermatology and Allergy, Clinical Research Center for Hair and Skin Science, Charité – Universitätsmedizin Berlin, Charitéplatz 1, 10117 Berlin, Germany; 2 University Centre for Nursing and Midwifery, Ghent University, Ghent, Belgium; 3 Institute of Clinical Trials Research, Clinical Trials Research Unit, University of Leeds, Leeds, UK; 4 Clinical Trials Research Centre (CTRC), North West Hub for Trials Methodology, University of Liverpool, Liverpool, UK; 5 School of Health Sciences, Nursing and Midwifery, University of Surrey, Guildford, UK; 6 School of Nursing and Midwifery, Royal College of Surgeons in Ireland, Dublin, Ireland; 7 School of Health Sciences, Örebro University, Örebro, Sweden; 8 School of Nursing & Midwifery, Menzies Health Institute Queensland, Griffith University and Gold Coast Hospital and Health Service, Southport, Qld Australia; 9 College of Nursing, University of Nebraska Medical Center, Omaha, NE USA; 10 Royal College of Surgeons in Ireland, Dublin, Ireland; 11 Monash University, Melbourne, Australia; 12 Faculty of Medicine and Health Sciences, Ghent University, Ghent, Belgium; 13 Lida Institute, Shanghai, China; 14 Cardiff University, Cardiff, Wales; 15 Faculty of Science, Quality of Life Office, School of Psychology, University of Sydney, Sydney, Australia; 16 Sydney Nursing School, Cancer Nursing Research Unit (CNRU), University of Sydney, Sydney, Australia; 17 Centre for Evidence-based Healthcare, Medical Faculty Carl Gustav Carus, Technical University Dresden, Dresden, Germany; 18 Institute for Social Medicine and Epidemiology, Nursing Research Unit, University of Lübeck, Lübeck, Germany

**Keywords:** Core domain set, Core outcome set, Pressure ulcer, Prevention, Scoping review, Delphi, Patient reported outcome, Clinical trial, Dermatology, Service user involvement

## Abstract

**Background:**

Core outcome sets (COS) are being developed in many clinical areas to increase the quality and comparability of clinical trial results as well as to ensure their relevance for patients. A COS represents an agreed standardized set of outcomes that describes the minimum that should be consistently reported in all clinical trials of a defined area. It comprises a core domain set (defining what core outcomes should be measured) and a core measurement set (defining measurement/assessment instruments for each core domain). For pressure ulcer prevention trials a COS is lacking. The great heterogeneity of reported outcomes in this field indicates the need for a COS.

**Methods/design:**

The first part of this project aims to develop a core domain set by following established methods, which incorporates four steps: (1) definition of the scope, (2) conducting a scoping review, (3) organizing facilitated workshops with service users, (4) performing Delphi surveys and establishing consensus in a face-to-face meeting with different stakeholders.

**Discussion:**

After achieving consensus on the core domain set, further work will be undertaken to determine a corresponding core measurement set. This will lead to better pressure ulcer prevention research in the future. There are a number of methodological challenges in the field of COS development. To meet these challenges and to ensure a high-quality COS, the OUTPUTS project affiliates to current standards and works in close collaboration with international experts and with existing international service user groups.

**Trial registration:**

The OUTPUTs project is registered in the COMET database: (http://www.comet-initiative.org/studies/details/283). Registered on 2015.

## Background

Results of clinical trials are critical for the development of evidence-based guidelines and decision-making at various levels of health care delivery. The selection of trial outcomes affects the validity, precision and clinical applicability of the trial findings. Trial outcomes should be biologically plausible, clinically meaningful and perceived as relevant from multiple perspectives, e.g. patients, informal caregivers, health professionals, policy-makers or manufacturers [1]. Further, these outcomes need to be measured with low risk of bias [2]. However, meta-epidemiological data indicate that outcomes and measurement methods often vary across trials for the same condition [3, 4], are of unclear clinical relevance [5, 6] or show selective reporting [3, 7]. Single trials often lack standards about the selection and measurement of outcomes and thus preclude meaningful summaries of evidence for a given health intervention.

To improve the rigour of single trials, and to facilitate meaningful evidence syntheses, standardising trial outcomes via the development of core outcome sets (COS) was recommended [8]. These are intended to represent “the minimum that should be measured and reported in all clinical trials of a specific condition” [9] and include a core set of outcome domains (core domain set) and a core set of measurement instruments to measure these outcomes (core measurement set) [10]. Thus, COS address questions about what to measure and how to measure it.

To date, COS have been developed for various medical conditions, such as rheumatologic conditions [11], hip fractures [12], atopic eczema [13] and incontinence-associated dermatitis [14]. An online database of COS projects has been established by the Core Outcome Measures in Effectiveness Trials (COMET) initiative [9], which is growing continuously [15]. The use of the COMET database is actively endorsed by the Cochrane Skin–Core Outcome Set Initiative (CS-COUSIN), which began in 2014 and comprises international, multidisciplinary researchers in the field of dermatology. The aim of this initiative is to support the development and to strengthen the quality of COS in dermatology, skin and soft tissue research by providing methodological advice, implementing tools for COS development and promoting exchange between the COS development groups in the field [16].

Pressure ulcers (PU) are lesions of the skin and underlying soft tissues caused by prolonged and/or elevated exposure to compressive and shearing forces. In the conceptual framework of Coleman et al. [17], immobility, impaired perfusion and skin/PU status (comprising existing and previous PU and general skin status) are proposed to be direct causal factors for PU development. There are a number of other indirect causal factors such as compromised sensory perception, diabetes mellitus, moisture and poor nutrition, which often occur in frail or critically ill people [17].

PUs are classified according to the visible depth of skin and tissue damage, ranging from non-blanchable erythema (category 1) to full thickness tissue loss involving muscle tissue and juxtaposed structures (category 4) [18]. Worldwide, the prevalence of PUs ranges from 0.3 to 46%, with highest prevalence in advanced age [19–21]. PUs can cause symptoms including pain [22–24], exudate and odour and compromise all areas of patient functioning, which consequently reduce quality of life [25, 26]. Moreover, patients with PUs are at high risk for further complications, such as nosocomial infections and sepsis, and they experience longer lengths of hospital stay [27]. Consequently, PUs represent a significant financial burden to healthcare organisations [28–30]. As PUs are considered to be largely preventable with good assessment and care, their occurrence is widely used as a quality indicator/patient safety parameter [31], and much effort is afforded to their prevention in clinical practice.

PU prevention comprises different interventions to reduce and/or eliminate the exposure of the patient’s skin to pressure, e.g. by allocation of pressure-redistributing support surfaces, regular repositioning and off-loading of highly vulnerable body sites, such as heels. Other preventive interventions target the promotion of the skin barrier, e.g. by good skin care, the reduction of shear and friction forces, e.g. via moving and handling activities, application of preventive dressings or the administration of nutritional supplementation.

An important outcome of PU prevention trials is the measurement of PU occurrence. However, substantial cross-trial variance and within-trial risk of bias have been noted with regard to this outcome measure [32, 33]. Trials show variations regarding PU classifications or methods used to determine the presence or absence of a PU (e.g. PU confirmation based on externally reviewed photographs or independent second skin inspection by one or multiple experts) [32]. In a meta-analysis by Shi et al., investigating the effect of support surfaces in reducing PU incidence, 57% of the included trials were rated as having serious or very serious limitations [34].

Furthermore, at present there is no consensus on how to report PUs. Often, measures of PU occurrence, such as incidence rates, include PUs across several categories (e.g. categories 1 to 4 or categories 2 to 4), thus combining superficial (category 1 and 2) and deep (category 3 and 4) PUs in one outcome measure. However, such combined estimates ignore the different etiological pathways of PU development and the targets of the preventive actions under experimental evaluation, e.g. interventions which mainly reduce friction forces may have a greater impact on superficial ulcers rather than deep ulcers [35].

Many other PU outcomes are also reported in trials, such as outcomes of pathophysiological changes, including tissue oxygenation and skin barrier changes, which have the potential to facilitate early prediction of a prevention strategy’s efficacy [36–39], or outcomes related to health care procedures and resource use. Patient-reported outcomes such as pain, experienced due to the preventive intervention or emerging before the development of a PU, are also important [40]. However, there are no agreed standards for which outcomes are of most importance and which measurement instruments or methods should be favoured. This not only complicates the synthesis of trial results, but also hinders the development of clear-cut evidence-based recommendations for PU prevention and, thus, evidence-based decision-making in clinical practice.

The overall aim of the Outcomes for Pressure Ulcer Trials (OUTPUTs) project is to improve methodological standards by developing a COS for the evaluation of the clinical efficacy, effectiveness and safety of PU prevention strategies. The focus of this protocol paper is the first part of this work, to develop a core domain set. The development of the corresponding core measurement set will be addressed in a separate publication.

## Methods/design

The process of this project follows general guidance and standards of COS development [10] and is part of the Cochrane Skin–Core Outcome Set Initiative [16]. This project complies with the methodological standards of this group [41] and also addresses the Core Outcome Set–Standards for Development (COS-STAD) recommendations by e.g. involving service users and other stakeholders during the consensus building process [42]. The core domain set will be developed by means of four main steps: (1) defining the scope, (2) conducting a scoping review, (3) organizing workshops with service users, and (4) undertaking Delphi surveys and establishing consensus at a face-to-face meeting with different stakeholders.

### Project management and stakeholders involved

All steps will be planned and led by a project team (Table 1). The members of the project team have experience in clinical and epidemiological studies, evidence summaries, structured consensus methods and guideline development in the field of PU prevention and/or in involving service users’ perspective in PU prevention research.

**Table 1:** Project management

Project team	Methodological Advisory Board
Members: • Balzer Katrin, Germany • Coleman Susanne, UK • Kottner Jan, Germany • Lechner Anna, Germany • Muir Delia, UK • Nixon Jane, UK	Members: • Bagley Heather, UK • Beeckman Dimitri, Belgium • Chaboyer Wendy, Australia • Cuddigan Janet, US • Moore Zena, Ireland • Rutherford Claudia, Australia • Schmitt Jochen, Germany
Tasks: • Identification of outcomes by performing a scoping review • Conducting workshops with service users to identify outcomes not captured by scoping review • Organizing Delphi surveys and a face-to-face meeting to find consensus on Core Domain Set	Tasks: • Methodological and content advice • Participating in Delphi surveys and face-to-face meeting

To ensure that all steps are properly addressed, a Methodological Advisory Board was established to provide methodological advice and guidance. Members are advanced experts of clinical research in PU prevention, COS development (e.g. representatives of the CS-COUSIN), evidence syntheses on PU prevention (e.g. representatives of the Cochrane Skin or Wound Groups), outcome assessment, service user involvement in COS development and statistics (Table 1).

Stakeholders participating in the Delphi surveys and the face-to-face meeting will incorporate service users, physicians and other health professionals representing various settings of health care, researchers, representatives of the health care management and manufacturers. Service users are people who have experience of being exposed to elevated PU risk and/or suffering from a PU, or have acted, or do currently act, as an informal caregiver for a person at risk or with existing PU [43]. Given the prominent role of patients’ perspective for the validity and clinical relevance of trial findings [44, 45], early and systematic involvement of service users as defined above is considered crucial for COS development [46, 47]. Therefore, representatives of the Pressure Ulcer Service User Network (PURSUN) [48] from Leeds (UK) will be regularly involved throughout the OUTPUTs project, e.g. by inviting them to service user workshops or to participate in Delphi surveys (see corresponding sections below).

### Scope specification

Through structured discussion within the project team and the methodological advisory board, the applicability of the intended COS was defined as follows: the COS should be applicable to all types of clinical trials investigating the clinical efficacy or effectiveness of all types of PU prevention interventions. The COS should be applicable to studies including adult patients aged ≥ 18 years. There should be no restriction concerning the health care setting or geographical areas.

### Scoping review

The project team will undertake a scoping review to identify and classify outcomes that may be important in clinical trials [49]. The main characteristic of a scoping review is that an overview of a broad topic is given, examining the extent and variety of the evidence on a topic. Compared to a systematic review, a scoping review allows a more general question and has less depth, but can cover a broader conceptual range [50, 51]. Systematic searches will be carried out in following electronic databases: Cochrane Wounds Group/Cochrane Skin Group Cochrane Wounds Group Specialised Register, Cochrane Central Register of Controlled Trials, Ovid MEDLINE, Ovid EMBASE, EBSCO CINAHL, PsychINFO, British Nursing Index, Allied and Complementary Medicine Database, Web of Knowledge, Clinical Trials.gov and the WHO International Clinical Trials Registry Platform Search Portal.

Any intervention claimed to prevent PU, including PU risk assessment, will be eligible for inclusion. This scoping review will include two information sources: (i) controlled trials and systematic reviews on the efficacy, effectiveness and/or safety of any PU prevention intervention; and (ii) primary studies and systematic reviews specifically exploring patient-reported outcomes related to PUs or PU prevention. In addition, full health economic evaluations and any kind of white or position papers on outcomes relevant to the science or practice of PU prevention will be included. Trials reporting PU-related outcomes only as a secondary or safety outcome of interventions which primarily target conditions other than PU risk will be excluded from this review. As the main objective of this review is to ideally cover the full scope of outcomes used in previous studies, and not to evaluate the quality of study results, no formal critical appraisal of study quality will be conducted. The target material will not be limited to specific health care settings or target populations. For feasibility reasons, only papers published in the English language will be included. No restrictions will be applied in terms of publication date, except for the primary studies on patient-reported outcomes, which will only be considered for inclusion if they were published after 2008, as the time period before that date is covered by a systematic review [25].

References will be imported into the Covidence platform (https://www.covidence.org/), which will be used for the study selection procedures. After removal of duplicates, the remaining references will be assessed for eligibility by two members of the project team using a two-step approach (title and abstract screening followed by full-text screening). Cases of disagreement will be solved within the project team through discussion.

Data extraction will be carried out by two members of the project team and cross-checked by another project team member. Among other characteristics the following details will be extracted: (a) bibliographic data (first author, year of publication, country), (b) study design, (c) setting, (d) type of interventions under evaluation, (e) outcomes used to assess the efficacy or effectiveness of the preventive measure, including the operational definition, if presented by the authors, as well as (f) safety and (g) economical outcomes. Outcomes will be extracted as presented in the primary studies and reviews.

Extracted outcomes will be classified into core areas and associated domains following a newly developed outcome classification system for COS [52]. A core area reflects “an aspect of health or health condition that needs to be measured” to assess the effects of a health intervention [53]. It is usually understood to represent this aspect at the most abstract level, e.g. physiological outcomes or life impact outcomes [52], comprising several domains which reflect the various dimensions of the condition of interest, e.g. with regard to the affected body structure, function or area of life impact. The project team will develop, based on the scoping review, a preliminary and classified longlist of domains considered relevant for PU prevention trials, i.e. a preliminary core domain set. Together with a complete overview of the review results, this preliminary core domain set will be presented to the Methodological Advisory Board for further discussion.

### Service user domain workshops

At least two facilitated workshops with service users will take place in Leeds, UK. These workshops serve a dual purpose. Firstly we aim to test out approaches to describing and discussing COS development within this group, which will inform our approach during the later stages of the project. Secondly we will begin to collect data around which outcomes are important from a service user perspective. Specifically we aim to explore service users’ views on the preliminary results of the scoping review, identify potentially relevant outcomes not identified via the scoping review and gain a greater understanding of why specified outcomes are important to service users. Participants of the workshops are from an established Pressure Ulcer Research Service User Network (PURSUN UK; http://www.pursun.org.uk/) who comprise patients and carers with direct experience of living with PU or PU risk.

PURSUN was set up to improve the quality of patient and public involvement (PPI) in PU research. Collectively the workshops provide the opportunity to capture a wide range of service user perspectives to inform the domains.

During the workshops, we will present an overview of the OUTPUTs project, explaining the aims, methods and preliminary results. This will be developed by an experienced PPI officer and presented in lay terms using an accessible format. It will be supported by information about COS highlighted in the COMET information leaflet ‘Involving patients and the public in improving research’ [54] to explain what an outcome is and how this could translate to PU prevention research. The group will then split into subgroups to discuss the service users’ understanding of each outcome/domain identified by the scoping review, its relevance for PU prevention trials and likely concerns about measurement. In order to clarify possible uncertainties and to record the conversation in written form, each subgroup discussion will be facilitated by a project member. After re-convening to the full group, further discussion will take place and this discussion will be audio recorded and transcribed. The researcher will listen to the audio tapes and read the transcripts to ensure completeness. The data will be coded with categories based on the domains identified in the scoping review, in keeping with directed content analysis [55]. Further codes can be added as they emerge from the data. A summary report of the meeting will be written and reviewed by the facilitators and service user participants to ensure it reflects group discussions. We will seek feedback from participants at the end of the session and at a separate PURSUN meeting. People will be asked to consider how well the project was explained, whether they felt able to contribute meaningfully and how best to gain service users perspectives throughout the Delphi survey and face to face consensus meeting.

### Delphi surveys

Delphi studies are an established method for formal consensus development [56], consisting of iterative surveys among a representative sample of stakeholders who are asked to express their views both quantitatively and qualitatively [57, 58]. Based on the results of the scoping review and the workshops with service users, an international Delphi study will be conducted over a minimum of two rounds in order to achieve preliminary consensus about the importance of outcome domains identified so far. A broad geographical coverage of participating stakeholders (service users, physicians and other health professionals, researchers, representatives of the health care management and manufacturers) will be targeted via links with existing professional and service user groups.

Prior to the first Delphi round, a draft version of the survey and supporting materials (e.g. overview of the review results) will be discussed with the service users group and the Methodological Advisory Board. Based on discussions, the survey and supporting material will be refined, followed by a pilot test with regard to the feasibility of the survey, involving members of the service user group.

Data will be collected via an online survey platform, ‘COMET Delphi Manager’ [59], allowing participants to rate the relevance of the core outcome domains identified in the previous project steps on a nine-step numeric rating scale, inspired by the GRADE working group: scores 1 to 3 indicate outcomes of limited importance, 4 to 6 indicate outcomes of non-critical importance, and 7 to 9 indicate outcomes of critical importance [60–62]. The presentation of the outcomes to the participants will be randomized in order to minimize risk of bias. The participants will have the possibility to add comments on each of the proposed outcomes. They will also be invited to list additional relevant outcomes not included in the survey.

The decision regarding which outcome should be included in the preliminary core domain set will follow a consensus definition already used in previous COS projects [13, 63] and favoured by the OMERACT (Outcome Measures in Rheumatology) initiative [53, 64]. According to this decision rule, each outcome may fall into one of three categories: (a) consensus that the outcome should be part of the COS (≥ 70% participants scoring 7 to 9 and ≤ 15% participants scoring 1 to 3); (b) consensus that the outcome should not be part of the COS (≥ 70% participants scoring 1 to 3 and ≤ 15% participants scoring 7 to 9); (c) no consensus (any other scoring distributions) [10]. After the first Delphi round, a summary of the stakeholder group’s scores and newly added outcomes will be presented to the participants. The summary will be reviewed by service users to ensure it is accessible and clear to lay people. The feedback of the results of Delphi round 1 gives the participants the opportunity to contemplate and revise their own opinions by rerating all outcomes in round 2. This may result in a better agreement between the participants [65, 66]. At the end of Delphi round 2 each outcome will be classified according to the consensus definition described above. All of the outcomes falling into category (a) will be included in the preliminary core domain set. Outcomes falling into category (b) will be excluded and thus will not be followed further. In case of outcomes falling into category (c) a third Delphi round will be conducted. At the end of each Delphi round, qualitative and quantitative data will be descriptively analyzed. Qualitative data analysis will follow established methods of descriptive analyses in qualitative research [67, 68]. All quantitative analyses will be conducted using IBM SPSS Statistics 23 software. For each outcome domain descriptive statistics will be determined: frequency distribution, median and interquartile range (IQR), both for the total sample and stratified per stakeholder group. The same weight will be attributed to each participant irrespective of the stakeholder group. To allow descriptive analyses, the following socio-demographic data will be gathered in the first Delphi round: (a) stakeholder group, to which the person belongs to (b) country, (c) age and gender, (d) highest degree of education, (e) experience in preventing PU.

### Face-to-face meeting

Upon completion of the Delphi surveys, a face-to-face meeting will be organized in order to reach final consensus about inclusion in the core domain set. The face-to-face meeting will be planned in accordance with COMET guidance [69] and principles established in previous consensus/servicer user engagement work [70] to ensure it is accessible to service users as well as professionals. This includes organizing the meeting well in advance, holding it at an accessible venue, using an expert facilitator to lead the meeting and ensuring support for service users before, during and after the meeting to facilitate their contribution [71]. The outcomes of unclear importance, and those rated as being critically important, will be subject to structured discussions. Participants of the Delphi surveys who have completed all rounds will be invited to join the face-to-face meeting, which will take place at the annual meeting of European Pressure Ulcer Advisory Panel in 2020. A summary of previous results, including an overview of how each stakeholder group has scored the relevant outcomes, will be presented. The participants will have the opportunity to discuss each outcome, exchange their views and convince others. After discussion, each outcome under debate will be scored by the participants anonymously with the nine-step numeric rating scale described above. To include an outcome in the core domain set, at least 70% of the participants need to score it as critically important [10, 72].

## Discussion

The planned project follows current guidance to develop a high-quality COS in the field of PU prevention and deals with the first stage on developing a core domain set. A number of methodological challenges in the field of COS development in general [73–76], and specifically for COS, which focus on prevention.

So far only a small number of COS exist for trials investigating preventive strategies [77–79]. In general it seems to be more complicated to define preventive effects compared to treatment effects. One challenge will be to ensure that all participants will have a correct understanding of the task. It may be challenging to make clear to service users and clinicians that the outcomes under discussion refer to clinical interventions with the major goal of “non-occurrence”, or prevention of deterioration. Confusion may be possible as a clear distinction between preventive and treatment measures cannot always be made. Many interventions targeting prevention of PUs, or preventing the deterioration of an existing PU, are also important for treatment, e.g. repositioning or the use of pressure-redistributing surfaces. This overlap between prevention and treatment interventions is described by other developers of COS for prevention trials as well [77, 80]. It is hoped that work in the preceding service user domain workshops will facilitate clarity about these potential areas of confusion. In addition it will be important to impart to the participants of the service user workshops and to the stakeholders taking part in the Delphi surveys that a COS aims to define outcomes for clinical trials research rather than for clinical practice.

Further, there is a range of different PU prevention strategies and the relevance of some outcomes is bound to the type of intervention. For example trials analyzing the administration of nutritional supplements often report the outcomes total serum protein or weight gain, which are outcomes plausible just for this type of intervention. It may be difficult to develop a COS which meets the complexity and diversity of PU preventive strategies as a whole. At the present time it is not entirely clear whether ‘generic’ core outcomes will be developed or whether the development of intervention-specific core outcomes will be required [41].

In general, there is discussion of how specified outcomes should be defined in a COS. Besides the determination of outcomes/domains and the corresponding measurement instrument, the time frame of the outcome measurement and the compilation of results (e.g. means, proportions) are important as well. Moreover, uncertainty remains about at what level of abstraction domains should be defined (e.g. adverse events in general versus a specific adverse event like falls or itching) [75]. To meet all these challenges properly it is important to follow the prescribed process of current standards step by step. One critical factor in this process is to include content and method experts and to integrate the experience of service users as well as of other stakeholders. Only a high-quality COS can improve the quality of trial results and promote evidence-based recommendations.

### Limitations

The aim of the OUTPUTs project is to develop a global COS. As the project team is Europe centred (Germany and UK), we endeavoured to widen the geographical distribution within the Methodological Advisory Board. However, representatives of Africa and Asia are missing. Thus, an effort will be made to ensure a wide geographical coverage within the Delphi surveys. As only papers in the English language will be included in the scoping review, a possible language bias may occur. Further, although the literature search will be conducted in most relevant electronic databases, including the largest trial registry database, some publications might not be identified.

### Future research plan

After the development of a core domain set, inquiries for reaching evidence-based consensus on preferred measurement tools for the defined core outcomes are planned. This step will consist of three stages: a systematic review, guided workshops with service users and a consensus-building process based on the Nominal group technique. The aim is the publication of a COS comprising a full list of included outcomes and of corresponding measurement instruments.

## Trial status

The development of the core domain set is active and ongoing in the phase of data extraction for the scoping review.

## Data Availability

Not applicable.

## Abbreviations

COMET Initiative: Core Outcome Measures in Effectiveness Trials Initiative
COS: Core outcome set
COSMIN: Consensus-based Standards for the Selection of Health Measurement Instruments
COS-STAD: Core outcome set–standards for development
CSG-COUSIN: Cochrane Skin Group Core Outcome Set Initiative
OMERACT initiative: Outcome Measures in Rheumatology initiative
OUTPUTs project: Outcomes for Pressure Ulcer Trials project
PU: Pressure ulcer
PURSUN: Pressure Ulcer Service User Network

## References

[1] Guyatt GH (2011). GRADE guidelines: 2. Framing the question and deciding on important outcomes. J Clin Epidemiol.

[2] Guyatt GH (2011). GRADE guidelines: 4. Rating the quality of evidence--study limitations (risk of bias). J Clin Epidemiol.

[3] Page MJ, et al. Bias due to selective inclusion and reporting of outcomes and analyses in systematic reviews of randomised trials of healthcare interventions. Cochrane Database Syst Rev. 2014;(10):Mr000035. 10.1002/14651858.MR000035.pub2. 10.1002/14651858.MR000035.pub2 PMC8191366 25271098

[4] Sharif MO (2015). A systematic review of outcome measures used in clinical trials of treatment interventions following traumatic dental injuries. Dent Traumatol.

[5] Gandhi GY (2008). Patient-important outcomes in registered diabetes trials. JAMA.

[6] Pino C (2012). Outcomes in registered, ongoing randomized controlled trials of patient education. PLoS One.

[7] Kirkham JJ (2010). The impact of outcome reporting bias in randomised controlled trials on a cohort of systematic reviews. BMJ.

[8] Clarke M (2007). Standardising outcomes for clinical trials and systematic reviews. Trials.

[9] COMET Initiative. http://www.comet-initiative.org/. Cited 5 June 2018.

[10] Williamson PR (2017). The COMET handbook: version 1.0. Trials.

[11] Tugwell P (2007). OMERACT: an international initiative to improve outcome measurement in rheumatology. Trials.

[12] Haywood KL (2014). Developing a core outcome set for hip fracture trials. Bone Joint J.

[13] Schmitt J (2015). The Harmonizing Outcome Measures for Eczema (HOME) roadmap: a methodological framework to develop core sets of outcome measurements in dermatology. J Invest Dermatol.

[14] Van den Bussche K (2018). Core outcome domains in incontinence-associated dermatitis research. J Adv Nurs.

[15] Davis K (2018). Choosing important health outcomes for comparative effectiveness research: An updated systematic review and involvement of low and middle income countries. PLoS One.

[16] Schmitt J (2016). Report from the kick-off meeting of the Cochrane Skin Group Core Outcome Set Initiative (CSG-COUSIN). Br J Dermatol.

[17] Coleman S (2014). A new pressure ulcer conceptual framework. J Adv Nurs.

[18] Haesler E, National Pressure Ulcer Advisory Panel, E.P.U.A.P.a.P.P.P.I.A (2014). Prevention and treatment of pressure ulcers: Quick reference guide.

[19] Hahnel E (2017). The epidemiology of skin conditions in the aged: A systematic review. J Tissue Viability.

[20] VanGilder C (2017). The International Pressure Ulcer Prevalence Survey: 2006-2015: A 10-year pressure injury prevalence and demographic trend analysis by care setting. J Wound Ostomy Continence Nurs.

[21] Tubaishat Ahmad, Papanikolaou Panos, Anthony Denis, Habiballah Laila (2017). Pressure Ulcers Prevalence in the Acute Care Setting: A Systematic Review, 2000-2015. Clinical Nursing Research.

[22] McGinnis E (2014). Pressure ulcer related pain in community populations: a prevalence survey. BMC Nurs.

[23] Gorecki C (2011). Patient-reported pressure ulcer pain: a mixed-methods systematic review. J Pain Symptom Manag.

[24] Briggs M (2013). The prevalence of pain at pressure areas and pressure ulcers in hospitalised patients. BMC Nurs.

[25] Gorecki C (2009). Impact of pressure ulcers on quality of life in older patients: a systematic review. J Am Geriatr Soc.

[26] Gorecki C (2010). Development of a conceptual framework of health-related quality of life in pressure ulcers: a patient-focused approach. Int J Nurs Stud.

[27] Moore Z (2013). US Medicare data show incidence of hospital-acquired pressure ulcers is 4.5%, and they are associated with longer hospital stay and higher risk of death. Evid Based Nurs.

[28] Dealey C (2012). The cost of pressure ulcers in the United Kingdom. J Wound Care.

[29] Schuurman JP (2009). Economic evaluation of pressure ulcer care: a cost minimization analysis of preventive strategies. Nurs Econ.

[30] Severens JL (2002). The cost of illness of pressure ulcers in The Netherlands. Adv Skin Wound Care.

[31] Kottner J (2018). Measuring the quality of pressure ulcer prevention: A systematic mapping review of quality indicators. Int Wound J.

[32] McInnes E, et al. Support surfaces for pressure ulcer prevention. Cochrane Database Syst Rev. 2015;(9):Cd001735. 10.1002/14651858.CD001735.pub5. 10.1002/14651858.CD001735.pub5 PMC7075275 26333288

[33] Moore ZEH, Webster J. Dressings and topical agents for preventing pressure ulcers. Cochrane Database Syst Rev. 2013;(Issue 8):CD009362. 10.1002/14651858.CD009362.pub2. 10.1002/14651858.CD009362.pub2 23955535

[34] Shi C (2018). Support surfaces for pressure ulcer prevention: A network meta-analysis. PLoS One.

[35] Kottner J, Gefen A (2012). Incidence of pressure ulcers as primary outcomes in clinical trials: a comment on McInnes et al. (2012). Int J Nurs Stud.

[36] Bader DL, Worsley PR (2018). Technologies to monitor the health of loaded skin tissues. Biomed Eng Online.

[37] Pfannes EKB (2018). Patterns and associations of structural and functional cutaneous responses during loading at heel and sacral skin in aged females: A reanalysis of clinical study data. J Tissue Viability.

[38] Dobos G (2015). Weight-bearing-induced changes in the microtopography and structural stiffness of human skin in vivo following immobility periods. Wound Repair Regen.

[39] Kottner J (2015). Skin response to sustained loading: A clinical explorative study. J Tissue Viability.

[40] Smith IL (2017). Exploring the role of pain as an early predictor of category 2 pressure ulcers: a prospective cohort study. BMJ Open.

[41] Kottner J (2018). Core outcome sets in dermatology: report from the second meeting of the International Cochrane Skin Group Core Outcome Set Initiative. Br J Dermatol.

[42] Kirkham JJ (2017). Core Outcome Set-STAndards for Development: The COS-STAD recommendations. PLoS Med.

[43] National Institute for Health Research. The James Lind Alliance Guidebook. Version 8. Novmeber 2018; Available from: http://www.jla.nihr.ac.uk/jla-guidebook/downloads/Version-8-JLA-Guidebook-for-download-from-website.pdf.

[44] Evans I, et al. So what makes for better healthcare? In: Thornton H, Chalmers I, Glasziou P, editors. Testing treatments: Better research for better healthcare. London: Pinter & Martin; 2011. p. 143–59. 22171402

[45] Chalmers I (2014). How to increase value and reduce waste when research priorities are set. Lancet.

[46] Macefield RC (2014). Developing core outcomes sets: methods for identifying and including patient-reported outcomes (PROs). Trials.

[47] Gargon E (2014). Choosing important health outcomes for comparative effectiveness research: a systematic review. PLoS One.

[48] Pressure Ulcer Research Service User Network. 5 June 2018; http://medhealth.leeds.ac.uk/info/2660/pressure_ulcer_research_service_user_network/1728/about_us.

[49] Peters MD (2015). Guidance for conducting systematic scoping reviews. Int J Evid Based Healthc.

[50] Peterson J (2017). Understanding scoping reviews: Definition, purpose, and process. J Am Assoc Nurse Pract.

[51] Tricco AC (2018). PRISMA Extension for Scoping Reviews (PRISMA-ScR): Checklist and explanation. Ann Intern Med.

[52] Dodd S (2018). A taxonomy has been developed for outcomes in medical research to help improve knowledge discovery. J Clin Epidemiol.

[53] Boers M (2014). Developing core outcome measurement sets for clinical trials: OMERACT filter 2.0. J Clin Epidemiol.

[54] COMET Initiative: Involving patients and the public in improving research. 25 Jan 2019; Available from: http://www.comet-initiative.org/assets/downloads/COMET%20Plain%20Language%20Summary%20v4.pdf.

[55] Hsieh H-F, Shannon SE (2005). Three approaches to qualitative content analysis. Qual Health Res.

[56] Dalkey N (1969). An experimental study of group opinion: The Delphi method. Futures.

[57] Nair R (2011). Methods of formal consensus in classification/diagnostic criteria and guideline development. Semin Arthritis Rheum.

[58] Sinha IP (2011). Using the Delphi technique to determine which outcomes to measure in clinical trials: recommendations for the future based on a systematic review of existing studies. PLoS Med.

[59] COMET Initiative Delphi Manager. Available from: http://www.comet-initiative.org/delphimanager/. Accessed 16 July 2019.

[60] Schunemann HJ (2009). GRADE: from grading the evidence to developing recommendations. A description of the system and a proposal regarding the transferability of the results of clinical research to clinical practice. Z Evid Fortbild Qual Gesundhwes.

[61] McMillan SS (2016). How to use the nominal group and Delphi techniques. Int J Clin Pharm.

[62] Schünemann H (2013). Handbook for grading the quality of evidence and the strength of recommendations using the GRADE approach.

[63] Tong A (2015). Standardised outcomes in nephrology - Haemodialysis (SONG-HD): study protocol for establishing a core outcome set in haemodialysis. Trials.

[64] Toupin-April K (2017). Toward the development of a core set of outcome domains to assess shared decision-making interventions in rheumatology: Results from an OMERACT Delphi Survey and Consensus Meeting. J Rheumatol.

[65] MacLennan S (2018). A randomized trial comparing three Delphi feedback strategies found no evidence of a difference in a setting with high initial agreement. J Clin Epidemiol.

[66] Brookes ST (2016). Three nested randomized controlled trials of peer-only or multiple stakeholder group feedback within Delphi surveys during core outcome and information set development. Trials.

[67] Sandelowski M (2000). Combining qualitative and quantitative sampling, data collection, and analysis techniques in mixed-method studies. Res Nurs Health.

[68] Sandelowski M (2001). Real qualitative researchers do not count: the use of numbers in qualitative research. Res Nurs Health.

[69] COMET Initiative: Tips for designing an accessible core outcome set consensus meeting. 25 Jan 2019; Available from: http://www.comet-initiative.org/assets/downloads/Tips%20for%20Designing%20an%20Accessible%20Core%20Outcome%20Set%20Consensus%20Meeting%20final%2026-10-17.pdf

[70] Nixon J (2015). Pressure UlceR Programme Of reSEarch (PURPOSE): using mixed methods (systematic reviews, prospective cohort, case study, consensus and psychometrics) to identify patient and organisational risk, develop a risk assessment tool and patient-reported outcome Quality of Life and Health Utility measures. PGfAR J.

[71] Muir D (2014). ‘It ain’t what you do it’s the way that you do it!’: Facilitation techniques for patient and public involvement.

[72] Harman NL (2015). The importance of integration of stakeholder views in core outcome set development: Otitis media with effusion in children with cleft palate. PLoS One.

[73] Gargon E (2017). Improving core outcome set development: qualitative interviews with developers provided pointers to inform guidance. J Clin Epidemiol.

[74] Kottner J (2018). Moving core outcome sets in dermatology forward. Br J Dermatol.

[75] Kottner J, Schmitt J (2018). Core outcome sets in dermatology: next steps. Br J Dermatol.

[76] Young B, Bagley H (2016). Including patients in core outcome set development: issues to consider based on three workshops with around 100 international delegates. Res Involv Engagem.

[77] Lamont TJ (2017). Core outcomes in periodontal trials: study protocol for core outcome set development. Trials.

[78] Meher S, Cuthbert A, Kirkham JJ, Williamson P, Abalos E, Aflaifel N, Bhutta ZA, Bishop A, Blum J, Collins P, Devane D, Ducloy‐Bouthors A‐S, Fawole B, Gülmezoglu AM, Gutteridge K, Gyte G, Homer CSE, Mallaiah S, Smith JM, Weeks AD, Alfirevic Z (2018). Core outcome sets for prevention and treatment of postpartum haemorrhage: an international Delphi consensus study. BJOG: An International Journal of Obstetrics & Gynaecology.

[79] Matvienko-Sikar Karen, Griffin Ciara, McGrath Niamh, Toomey Elaine, Byrne Molly, Kelly Colette, Heary Caroline, Devane Declan, Kearney Patricia M. (2018). Developing a core outcome set for childhood obesity prevention: A systematic review. Maternal & Child Nutrition.

[80] Shireen M, et al. Core outcome sets for prevention and treatment of postpartum haemorrhage: an international Delphi consensus study. BJOG Int J Obstet Gynaecol. 2019;126(1):83–93. 10.1111/1471-0528.15335 29920912

[81] EPUAP Research Project Collaboration Funding. 18 Jan 2019; Available from: http://www.epuap.org/research-funding/#epuapfunding.

